# Effectiveness of a Single Fixed Dose of 3 mg Rasburicase for the Prevention and Management of Hyperuricemia in Tumor Lysis Syndrome in Adults With Cancer

**DOI:** 10.7759/cureus.68664

**Published:** 2024-09-04

**Authors:** Sabirin Bakhsh, Mansoor Ahmed Khan, Majed Alshamrani, Roula Mufti, Anjum Naeem, Mubarak AlMansour, Ahmad Alsaeed, Mousa Alahmari, Mohammed Aseeri

**Affiliations:** 1 Pharmaceutical Care Services, King Abdulaziz Medical City, Ministry of National Guard Health Affairs, Jeddah, SAU; 2 Medical Affairs, Johnson &amp; Johnson Innovative Medicine, Riyadh, SAU; 3 Oncology, King Faisal Specialist Hospital and Research Centre, Jeddah, SAU; 4 Adult Medical Oncology, Princess Noorah Oncology Center, King Abdulaziz Medical City, Ministry of National Guard Health Affairs, Jeddah, SAU; 5 Adult Hematology/Bone Marrow Transplantation (BMT), Princess Noorah Oncology Center, King Abdulaziz Medical City, Ministry of National Guard Health Affairs, Jeddah, SAU

**Keywords:** 3 mg single fixed dose, cost-effectiveness, hematology and oncology, rasburicase, tumor lysis syndrome

## Abstract

Background: Tumor lysis syndrome (TLS) is a critical and potentially fatal complication linked to specific types of cancer. Rasburicase stands as a crucial medication necessary for the prevention and treatment of hyperuricemia, a condition commonly associated with TLS. Due to a shortage of rasburicase during the COVID-19 pandemic, a fixed-dose strategy of 3 mg rasburicase was used in many adult cancer patients in our center.

Objective: The objective of this study was to assess the effectiveness of a fixed dose of 3 mg rasburicase in preventing and managing hyperuricemia associated with TLS in adult cancer patients.

Methods: We conducted a retrospective, observational cohort study between March 2020 and February 2022. The study included adult patients who received a fixed dose of 3 mg rasburicase. The primary outcome measure was the reduction in serum uric acid (UA) levels at 24 and 48 hours after treatment, with the aim of achieving and maintaining normal UA levels.

Results: Seventeen patients in the treatment group and 20 patients in the prevention group were included. In the treatment group, 15 (88%) patients had normalization of serum UA, which is considered to be <7 mg/dL (median: 4.48 mg/dL) at 24 hours, and 16 (94%) patients had achieved normal UA at 48 hours (median: 2.78 mg/dL) after receiving the rasburicase dose. In the prevention group, all 20 (100%) patients achieved normal UA at 24 hours after receiving the rasburicase dose.

Conclusion: Based on these findings, a single fixed dose of 3 mg rasburicase is effective for preventing and managing hyperuricemia associated with TLS in high-risk patients.

## Introduction

Tumor lysis syndrome (TLS) is a life-threatening complication that can arise in patients with certain types of cancer. This condition occurs when tumor cells undergo breakdown, leading to the sudden release of intracellular contents into the bloodstream. This release can be triggered by cytotoxic treatments or occur spontaneously. It leads to various electrolyte and laboratory changes, such as hyperuricemia, hyperkalemia, hyperphosphatemia, and hypocalcemia. These abnormal laboratory findings can have serious consequences, including cardiac arrhythmias, acute kidney injury, seizures, and even death if not promptly treated. The clinical signs of TLS typically appear 12-72 hours after beginning chemotherapy treatment [1-5]. Cairo and Bishop defined laboratory TLS in adults as the presence of two or more specific metabolic abnormalities. These abnormalities include uric acid (UA) levels of 476 mmol/L (8 mg/dL) or a 25% increase from baseline, potassium levels of 6.0 mmol/L or a 25% increase from baseline, phosphorus levels of 1.45 mmol/L (4.5 mg/dL) or a 25% increase from baseline, and calcium levels of 1.75 mmol/L (7 mg/dL) or a 25% decrease from baseline. The clinical definition of TLS includes the following criteria: (1) creatinine levels that are 1.5 times the upper limit of normal (ULN), adjusted for age (>12 years); (2) presence of cardiac arrhythmia or sudden death; and (3) occurrence of seizures [3].

The current treatment for TLS involves several approaches, including intensive hydration, forced diuresis, and the administration of medications such as allopurinol. Allopurinol is a type of xanthine oxidase inhibitor that works by preventing the formation of UA. Another medication used for TLS treatment is rasburicase, which is a recombinant form of urate oxidase. Rasburicase facilitates the conversion of UA into allantoin, leading to increased levels of urinary excretion. It is important to note that while rasburicase aids in the elimination of UA, it does not directly inhibit its formation [5]. Rasburicase was approved by the United States Food and Drug Administration (US FDA) for adults in 2009, which helped in the prevention and management of hyperuricemia of TLS in rapidly proliferating cancers. The approved dosage of rasburicase is 0.2 mg/kg, administered intravenously once daily for five days, within 4-24 hours of chemotherapy [6]. Several published guidelines suggest using 0.2 mg/kg for one to seven days [7-10].

In a retrospective single-center chart review study conducted by Khan et al., the effectiveness of a single fixed dose of rasburicase (6 mg) was evaluated in 94 adult patients for the prevention or management of hyperuricemia associated with TLS. The study found that 79% (75) of the patients achieved normalization of serum UA levels (<7 mg/dL), with a median of 1.00 mg/dL, within 24 hours of receiving the treatment. Approximately 81% (77) of the patients achieved normalized UA levels within 48 hours, with a median of 1.96 mg/dL. These normal UA levels, with a median of 2.97 mg/dL, were maintained for up to 96 hours. The analysis also revealed significant cost savings of $320,000 in the treatment of these patients using the single fixed dose of rasburicase compared to the multiple-day dosing recommended by the FDA. This indicates that the single fixed dose approach is cost-effective and may provide clinical benefit [11].

In a study by Marjoncu and Holman, 79 adult cancer patients who received rasburicase were analyzed to compare the rates of UA normalization within 24 hours using a 3 mg dose versus a 6 mg dose. The results showed no significant difference in UA normalization between the two groups, with 95% of patients in the 3 mg group and 82% in the 6 mg group achieving normalization. Additionally, implementing the lower dose led to over $300,000 in annual cost savings. The researchers concluded that a 3 mg dose of rasburicase effectively treats hyperuricemia within 24 hours and offers significant cost savings [12].

Princess Noorah Oncology Center (PNOC) is one of the largest tertiary care referral oncology facilities in the Western Province of Saudi Arabia. It plays a crucial role in treating various types of cancer, which are commonly associated with TLS. However, the COVID-19 pandemic has had a negative impact on medical care, including challenges with pharmaceutical care and procurement. Consequently, the availability of rasburicase had been affected. To address this issue, PNOC had implemented a policy during the COVID-19 pandemic that involves the use of a single fixed dose of 3 mg rasburicase for preventing or managing hyperuricemia in high-risk TLS patients as per our institutional TLS guidelines.

To our knowledge, only a few studies have examined the effectiveness of a single fixed dose of 3 mg rasburicase. We planned to conduct this retrospective study to evaluate the effectiveness of this treatment for preventing and managing hyperuricemia in adult cancer patients with TLS. Additionally, we aimed to determine the cost-saving impact of using a single fixed dose of 3 mg rasburicase.

This article was previously posted to the Pharmacy MDPI preprint server on May 14, 2024 (https://www.preprints.org/manuscript/202405.0962/v1). The results of this study were presented as posters at two international meetings: the Hematology Oncology Pharmacy Assembly Meeting in Phoenix, USA (March 28-April 1, 2023) and the Third Saudi Society of Clinical Pharmacy Meeting in Riyadh, Saudi Arabia (September 7-9, 2023). An oral presentation was also delivered at a local meeting "Saudi Pharmacy Resident Day" organized by the Saudi Commission for Health Specialists in August 2023. The results were shared with our oncology department as well as oncologists and hematologists in the country.

## Materials and methods

This is a retrospective, observational cohort single-center study conducted between March 2020 and February 2022 according to the guidelines of the Declaration of Helsinki and was approved by the Institutional Review Board of King Abdullah International Medical Research Center, Saudi Arabia, with IRB approval number IRB/1259/22 on July 18, 2022. 

Our primary objective was to assess the effectiveness of a single fixed dose of 3 mg rasburicase in lowering uric levels to 420 mmol/L (<7 mg/dL) from baseline at 24 hours and 48 hours after administration. Our secondary objectives were to determine the effectiveness of a single fixed dose of 3 mg rasburicase in lowering UA levels to <7 mg/dL at 72 hours and 96 hours after administration; to lower serum creatinine level from baseline to 132 µmol/L (<1.5 mg/dL) at 96 hours after administration; to determine the number of rasburicase doses required for the prevention or treatment of hyperuricemia associated with TLS; and to determine the cost-saving impact of using a single fixed dose of 3 mg rasburicase.

We examined the electronic medical records (EMRs) at PNOC for eligible adult patients aged over 14 years (the Saudi healthcare system defines adults as those older than 14 years) with a confirmed diagnosis of any type of cancer who received one or more fixed doses of 3 mg rasburicase for the treatment or prevention of hyperuricemia between March 2020 and February 2022. Patients with serum UA levels above 7 mg/dL were considered to have hyperuricemia, while acute renal dysfunction was defined as serum creatinine levels above 132 µmol/L (1.5 mg/dL).

Patients who did not have serum UA and serum creatinine levels measured before and after rasburicase administration or who received any different dose of rasburicase were excluded from the study. Patients were classified into two categories based on indication (treatment vs. prevention) considering UA level before rasburicase administration. Patients were included in the treatment group if their UA level before rasburicase administration was above 7 mg/dL, based on our institutional TLS guidelines. On the other hand, patients with UA levels below 7 mg/dL before rasburicase administration having no other laboratory abnormalities were included in the prevention group. Then, each category was described independently.

Demographic data, concurrent medication use, and laboratory parameters were collected for each patient in the study. UA levels were collected after rasburicase administration, with repeated measurements taken up to 96 hours after administration. To ensure accurate UA measurements, our institution followed specific handling procedures recommended in our center's TLS guidelines. These guidelines included collecting blood into prechilled tubes with heparin anticoagulant and immediately immersing and maintaining the samples in an ice water bath. Plasma samples were assayed within four hours of collection as per our TLS guidelines. The study also assessed the cost-saving impact of a single fixed dose of 3 mg rasburicase (used off-label) in comparison with the FDA-approved dose of 0.2 mg/kg IV daily for five days, using the actual weight of the patient included in the study to calculate cost savings.

Data collection and analysis

In our study, we followed several steps to collect data. We utilized a data collection sheet to document various variables and laboratory values for each patient. The recorded variables and laboratory values included patient demographics (age, sex, weight, height), type of cancer, medications received (rasburicase, allopurinol), serum UA levels, serum creatinine levels, serum lactate dehydrogenase (LDH) levels, serum potassium levels, serum phosphorus levels, and corrected serum calcium levels. These measurements were taken at baseline (pre-rasburicase administration) and at various time points post-rasburicase administration (24, 48, 72, and 96 hours). Patient charts in EMRs were accessed to obtain patient demographics, pertinent medication, and laboratory values using BestCare 2.0A, which is our electronic hospital information system. Data were saved and analyzed using Microsoft Office Excel 2010, protected by a password key to ensure the confidentiality of data. Serum UA levels were assessed at baseline (pre-rasburicase administration) and at 24, 48, 72, and 96 hours post-rasburicase administration. Serum creatinine, serum potassium, serum phosphorus, corrected serum calcium, and serum LDH were collected retrospectively, and the cost impact was calculated. The mean and standard deviations were reported for continuous variables; numbers and percentages were reported for nominal or categorical data.

## Results

We searched 254 patient charts of those who received any dose of rasburicase during the study period between March 2020 and February 2022 and found that only 37 patients who received a dose of 3 mg rasburicase and met the inclusion criteria were assigned to the study. Assigned patients were divided into two groups: treatment and prevention, based on baseline serum UA at the time of rasburicase dose administration. We assigned 17 (46%) patients to the treatment group and 20 (54%) patients to the prevention group.

Baseline characteristics of the study population

Table 1 provides the baseline characteristics of the treatment group (N=17), with a mean age of 55 years, and the prevention group (N=20), with a mean age of 50 years. Non-Hodgkin's lymphoma was the most common diagnosis in both the treatment and prevention groups.

**Table 1:** Baseline characteristics of the treatment and prevention groups (N=37).

Baseline characteristics	Treatment group (N=17)	Prevention group (N=20)
Age, mean (SD) years	55 (19)	50 (20.6)
Gender N (%)	Male 9 (52%)	Male 9 (45%)
	Female 8 (48%)	Female 11 (55%)
Weight mean (SD), kg	77 (30)	76 (33)
Diagnosis	Non-Hodgkin's lymphoma 10 (59%)	Non-Hodgkin's lymphoma 19 (95%)
	Others 7 (41%)	Others 1 (5%)

Baseline laboratory parameters

Table 2 describes the baseline laboratory parameters of patients in both the treatment and prevention groups. Our findings showed that three of the six laboratory parameters, including serum UA, serum creatinine, and serum LDH, were abnormally elevated in the treatment group, possibly indicating that the patients had TLS. Whereas only LDH was elevated in the prevention group.

**Table 2:** Baseline laboratory parameters of patients in the treatment and prevention groups (N=37). LDH: lactate dehydrogenase; UA: uric acid.

Baseline laboratory parameters of patients in treatment group (N=17)		
Baseline laboratory parameters	Range	Median
Serum UA, mmol/L	432-1229 (7.2-20.6 mg/dL)	608.5 (10.2) mg/dL
Serum LDH, U/L	143-1816	453
Serum creatinine, µmol/L	34-755 (0.38-8.54 mg/dL)	172 (1.94 mg/dL)
Serum potassium, mEq/L	3.4-6	4.1
Serum phosphorus, mmol/L	0.84-2.36 (2.6-7.3 mg/dL)	1.27 (3.39 mg/dL)
Adjusted calcium, mmol/L	1.78-2.34 (7.12-9.36 mg/dL)	2.19 (8.76 mg/dL)
Baseline laboratory parameters of patients in prevention group (N=20)		
Baseline laboratory parameters	Range	Median
Serum UA, mmol/L	82-394 (1.37-4.45 mg/dL)	194 (3.26 mg/dL)
Serum LDH, U/L	186-5666	615
Serum creatinine, µmol/L	42-330 (0.47-3.73 mg/dL)	64.5 (0.72 mg/dL)
Serum potassium, mEq/L	3.9-5.0	4.2
Serum phosphorus, mmol/L	0.66-1.67 (2.04-5.17 mg/dL)	1.05 (3.15 mg/dL)
Adjusted calcium, mmol/L	2.12-2.46 (8.48-9.84 mg/dL)	2.24 (8.96 mg/dL)

Table 3 displays the normalization of UA levels at 24, 48, 72, and 96 hours in both the treatment and prevention groups. We reported all serum UA levels that were obtained pre- and post-rasburicase for up to 96 hours. At 24 hours from receiving the rasburicase dose, 15 (88%) patients in the treatment group achieved normal UA level (<7 mg/dL). The remaining patients in the treatment group achieved normal UA level within 72 hours. Whereas all 20 (100 %) patients in the prevention group maintained normal UA (<7 mg/dL) level for 96 hours. Normalization of serum creatinine below 1.5 mg/dL was achieved in 90% patients in the prevention group, with a median of 0.62 mg/dL (range 0.39-3.29 mg/dL), and in 65% of the patients in the treatment group, with a median of 0.97 mg/dL (range 0.39-3.72 mg/dL) at 96 hours after rasburicase administration.

**Table 3:** Patients who achieved normal UA levels in the treatment and prevention groups. UA: uric acid.

Patient achieved normal UA level in treatment group				
Time	24 hours	48 hours	72 hours	96 hours
N (%)	15 (88%)	16 (94%)	17 (100%)	17 (100%)
Median UA (mg/dL)	4.48	2.78	3.31	3.79
Range (mg/dL)	Below 1-6.26	Below 1-6.85	Below 1-5.04	Below 1-5.05
Patient achieved normal UA level in prevention group				
Time	24 hours	48 hours	72 hours	96 hours
N (%)	20 (100%)	20 (100%)	20 (100%)	20 (100%)
Median UA (mg/dL)	1.61	1.08	1.49	1.89
Range (mg/dL)	Below 1-3.39	Below 1-4.82	Below 1-3.05	Below 1-4.8

Table 4 describes the total number of 3 mg fixed doses required to lower serum UA in both the treatment and prevention groups. In the treatment group, 16 (94 %) patients required a single 3 mg fixed dose of rasburicase to achieve normal UA levels (<7 mg/dL), whereas one (6%) patient required three doses of 3 mg fixed dose of rasburicase. While in the prevention group, 17 (85%) patients required single 3 mg fixed dose of rasburicase, two (10%) patients required two doses of 3 mg fixed dose of rasburicase, and only one (5%) patient required three doses of 3 mg fixed dose of rasburicase to maintain normal UA levels (<7 mg/dL).

**Table 4:** Total number of fixed dose/s of 3 mg rasburicase to lower UA in the treatment and prevention groups. UA: uric acid.

Total number of fixed dose/s of 3 mg rasburicase to lower UA	Number of patients in treatment group; n (%)	Number of patients in prevention group; n (%)
One dose	14 (82%)	19 (95%)
Two doses	2 (12%)	0
Three doses	1 (6%)	1 (5%)

Subgroup analysis

During our study, we identified patients with serum UA levels above 7 mg/dL within 96 hours of receiving the initial dose of rasburicase. These patients were considered potential candidates for a repeated dose. In the treatment group, three patients (15% of the group) received a repeat dose and only one patient was clinically indicated to receive the repeat dose and two patients received repeat dose unnecessarily. However, in the prevention group, only one patient (5% of the group) received an unnecessary repeated dose of rasburicase because their serum UA levels remained below the normal limit set by our institution for the entire 96-hour period. Overall, of the 37 patients, four (10.8%) received a repeated 3-mg dose of rasburicase. However, based on our findings, we determined that only one out of the four patients actually required the repeated dose. This indicates that the repeated dose was unnecessary in three patients.

Cost-saving benefit

As a single fixed dose of 3 mg rasburicase is an off-label dosing, we compared the cost of a single fixed dose of 3 mg rasburicase with FDA-approved dosing of the rasburicase for the same indication, i.e., 0.2 mg/kg IV daily for five days, using the actual weight of the patient included in the study to calculate the impact on the cost saving. The Saudi Food and Drug Authority (SFDA) cost of the rasburicase is 979.75 SAR (261.26 USD) per 1.5 mg vial. The cost of the institutional recommended dose of 3 mg during the COVID-19 pandemic was 979.75 × 2 vials = 1959.5 SAR (522.53 USD), whereas the cost of rasburicase therapy as per US FDA-recommended dose/schedule is much higher. The median weight in our study was 77 kg. Using US FDA-recommended dose of 0.2 mg/kg for five days would require 50 vials per patient (77 kg × 0.2 mg = 15.4 mg per day, which was rounded to 10 vials × 5 days = 50 vials). The cost of the 50 vials of the rasburicase is 50 × 979.75 = 48987.5 SAR (13063.33 USD), which means we potentially saved 48987.5 - 1959.5 = 47,028 SAR (12540.8 USD) per patient. The total cost saving using a single fixed dose of 3 mg rasburicase in 37 patients was 1,740,036 SAR (464,009 USD).

## Discussion

In our study, we observed that serum UA and serum creatinine levels remained stable and low for 24-96 hours. Around 88% of patients in the treatment group achieved normalization of UA within 24 hours, with all patients reaching normal levels within 72 hours. This reduction was sustained until the 96-hour mark in both the treatment and prevention groups. Patients who received a single fixed dose of 3 mg were monitored for TLS and had their UA levels documented daily for up to 96 hours. If the serum UA level remains above 7 mg/dL during this period, repeat dosing is considered as per our institutional TLS guidelines. Also, we found that out of the 37 patients, around 10.8% (four patients) received more than one 3-mg dose of rasburicase. However, our analysis showed that only one out of these four patients actually required the additional dose, indicating that it was unnecessary in three of them.

Rasburicase is a recommended treatment for managing or preventing hyperuricemia (high UA levels) in patients at high risk for TLS. This medication effectively reduces UA levels in individuals with TLS-related hyperuricemia [4,8]. Allopurinol, a xanthine oxidase inhibitor, is an alternative option for managing or preventing hyperuricemia associated with TLS. However, it is typically used in cancer patients with a low or intermediate risk of developing TLS [13]. It is crucial to screen patients for glucose-6-phosphate dehydrogenase deficiency (G6PD) before administering rasburicase. G6PD deficiency is a contraindication for the use of rasburicase because patients with this deficiency may have severe hemolytic anemia within two to four days after rasburicase initiation [14]. The US FDA initially approved rasburicase in 2002 to prevent or treat the hyperuricemia of TLS in children receiving chemotherapy who are at high risk for TLS [3,6]. Later on, the FDA granted approval for the use of rasburicase in the initial management of TLS in adults [6], based on a randomized phase 3 study of patients with hematologic malignancies at risk for hyperuricemia and TLS [15]. The FDA recommends a dose of 0.2 mg/kg of rasburicase given intravenously once daily for up to five days for the prevention or management of hyperuricemia associated with TLS in adult cancer patients. Many guidelines also support this dosing regimen, suggesting the use of 0.2 mg/kg for one to seven days [8,13,16]. However, several published studies have shown that lower doses of rasburicase, ranging from 3 to 7.5 mg as a single dose, can be equally effective in achieving desired outcomes and may result in cost savings [17,12,18].

The results of our new study are similar to the findings of our previously published study, “Effectiveness of a single 6-mg fixed dose of rasburicase for prevention or management of hyperuricemia associated with tumor lysis syndrome in adults with cancer” [11]. Based on the findings of the previous study, we recommended using the single 6 mg fixed-dose strategy for the prevention or management of hyperuricemia associated with TLS in our institution and others. We also recommended randomized, prospective studies to compare the use of a single fixed dose of 3 mg rasburicase versus a single fixed dose of 6 mg rasburicase and compare them to the FDA-approved dose of rasburicase to determine the optimal dosing schedule [11]. Moreover, our current study demonstrated that using a single fixed dose of 3 mg rasburicase resulted in significant cost savings compared to the multiple-day dosing regimen recommended by the FDA. The findings of this study confirm the effectiveness of lower doses of rasburicase used in the prevention or management of TLS in previously published studies [12,19].

The limitations of this study included its small sample size, having no comparative group, and being conducted at a single center in a retrospective fashion. Moreover, descriptive statistics were used to analyze the data.

## Conclusions

Our study has shown that administering a single fixed dose of 3 mg rasburicase effectively prevents or manages hyperuricemia in high-risk patients and maintains reduced UA levels for up to 96 hours. Additionally, this fixed-dose strategy is considered cost-effective. Based on these findings, we conclude that a single dose of 3 mg rasburicase, used with close monitoring, is sufficient to treat or prevent hyperuricemia associated with TLS in most adult cancer patients.
